# NH_4_F-assisted one-pot solution synthesis of hexagonal ZnO microdiscs for efficient ultraviolet photodetection

**DOI:** 10.1098/rsos.180822

**Published:** 2018-09-12

**Authors:** Borui Li, Kai Zhou, Zhao Chen, Zengcai Song, Dong Zhang, Guojia Fang

**Affiliations:** Key Lab of Artificial Micro- and Nano-Structures of Ministry of Education of China, School of Physics and Technology, Wuhan University, Wuhan 430072, People's Republic of China

**Keywords:** F-doped ZnO, ultraviolet photodetection, crystal growth

## Abstract

One-pot solution method to grow large hexagonal ZnO microdiscs with the aid of ammonium fluoride (NH_4_F) mineralizer has been realized. The size, morphology, crystallinity and optical properties of the synthesized ZnO microdiscs can be efficiently modulated by the concentration of NH_4_F. X-ray diffraction and scanning electron microscopy analyses illustrate that hexagonal ZnO microdiscs achieved at 0.03 M NH_4_F concentration have larger disc size and narrower full-width value at half maximum of (002) peak. It implies better crystal quality compared with those from other additive concentrations. Photoluminescence results also demonstrate the same trend. These results indicate that with proper addition of NH_4_F, the crystal quality of ZnO microdiscs has been improved and defects have been suppressed. Furthermore, a UV photodetector has been fabricated by simply transferring the ZnO microdiscs grown with 0.03 M NH_4_F onto a *p*-type silicon substrate. The device exhibits photosensitive behaviour at 365 nm UV light illuminating when −0.6 V is applied. The response time as well as recovery time is less than 0.1 s. The relatively large photoresponsivity of 1.19 A W^−1^ with power consumption less than 10 nW makes it possible in application field of highly efficient low power consumption UV detection.

## 1. Introduction

With a wide direct band gap of 3.37 eV and a large exciton binding energy of 60 meV, zinc oxide (ZnO) has received considerable attention in revealing the growth mechanism of its nanostructures. Before applying in such fields, revealing the growth mechanism of its various nanostructures (nanowires [1], nanotubes [2], nanobelts [3], nanoflowers [4], etc.) seems to be of more importance [5,6]. Most of the ZnO nanostructures used in optoelectronic devices are nanorod-like [7–9]. Owing to the high Q-factors, however, ZnO nanostructures such as micro-/nano-discs or plates, with suppressed c-axis orientation, are emerging and regarded as important building blocks for nanoscale optoelectronic devices [10]. Various fabrication methods have been reported to fabricate ZnO microdisc structure [11–13]. In order to obtain high-quality ZnO microdiscs, vapour phase methods, such as vapour phase transport (VPT) and chemical vapour deposition (CVD), have been widely used. The photoluminescence (PL) properties of ZnO nano-/micro-discs with whispering-gallery mode [14,15] and the growth mechanism have been studied [13,16]. However, the VPT and CVD methods need high temperature in the process, leading to high energy cost, which is not good for our environment. A simple and economic solution method with low growth temperature should be more favourable. The ZnO microdiscs obtained through solution method always have small size in diameter; low crystal quality is reported in the literature. Such small ZnO discs are very difficult to use in devices for optoelectronic devices [17,18]. Furthermore, single ZnO microdisc-based ultraviolet (UV) photodetectors are seldom reported. Thus, large ZnO microdiscs with excellent crystal quality are highly expected. Ammonium fluoride (NH_4_F) is an acidic mineralizer and is reported to play an important role in promoting the growth rate and improving the crystal quality of GaN and ZrO_2_ in the ammonothermal growth method [19–22]. Several F-doped ZnO films are realized through magnetron sputtering or pulsed laser deposition [23–25]. However, rare work is shown in the growth of ZnO nano- or micro-disc structures [26].

In this work, we report a simple one-pot solution method for the synthesis of large ZnO hexagonal microdiscs with the assistance of NH_4_F at a relatively low temperature (95°C). We found that NH_4_F concentration significantly affects the ZnO growth process. The morphology and crystal quality of ZnO microdiscs also vary with the NH_4_F concentration. With proper NH_4_F concentration, the diameter of ZnO hexagonal microdiscs can be as large as 16 µm. The growth rate and crystal quality of ZnO microdiscs are improved by adding a suitable amount of NH_4_F in the nutrient solution. A ZnO microdisc/*p*-Si heterojunction diode has been fabricated by transferring the individual ZnO microdisc grown with the additive of 0.03M NH_4_F onto a *p*-Si substrate for ultraviolet photodetection. The device exhibits good rectifying characteristic (approx. 245 at ± 1 V). The response time and recovery time is less than 0.1 s under UV illumination (365 nm, 4.1 mW cm^−2^). Furthermore, the device has shown good reproducibility with a large responsivity of about 1.19 A W^−1^, while the power consumption is less than 10 nW.

## 2. Experimental section

All reagents in this work are of analytical grade and used without further purification. The ZnO microdiscs were synthesized via a facile one-pot solution process. The precursor solution contained an aqueous solution of 10 mM zinc nitrate hexahydrate (Zn(NO_3_)_2_·6H_2_O), 10 mM hexamethylenetetramine (HMT), 1 mM sodium citrate (Na_3_C_6_H_5_O_7_) and different amounts of NH_4_F (0, 0.01, 0.03 and 0.05 M). The sodium citrate was frequently used as a surfactant to control the shape of ZnO crystals. The as-prepared solution was transferred to sealed glass jars for solution treatment at 95°C for 7 h in an oven. After that, the glass jars were cooled down to room temperature (RT) naturally and ZnO microdiscs were formed in the solution. Then, the obtained solution containing the ZnO microdiscs was dropped onto a clean *p*-Si substrate (1–10 Ω cm). The samples were annealed at 500°C in air for 6 h to form ZnO microdisc/*p*-Si heterojunction UV detectors. Finally, the In-Ga alloy was scraped onto the backside of the *p*-Si substrate as anode and the probe was directly pressed onto the microdisc as cathode. All the measurements were applied at RT.

The morphology of the ZnO microdiscs was characterized by a scanning electron microscopy (SEM, JEOL JSM-6700F). The crystal structure and phase composition were characterized by X-ray diffraction (XRD, Bruker AXS, D8 Advance). The photoluminescence (PL) measurements were carried out under a 325 nm He-Cd laser at RT and the emission was collected via a HORIBA Jobin-Yvon monochromator. Raman spectra were collected through a HORIBA Jobin-Yvon LabRam HR system equipped with a 488 nm laser. The current–voltage (*I*–*V*) curves were measured by a Keithley 4200 semiconductor parameter analyser. The photoresponsivity was measured by a home-built testing system, with a xenon light source and a spectrometer.

## 3. Results and discussion

**Figure 1.**
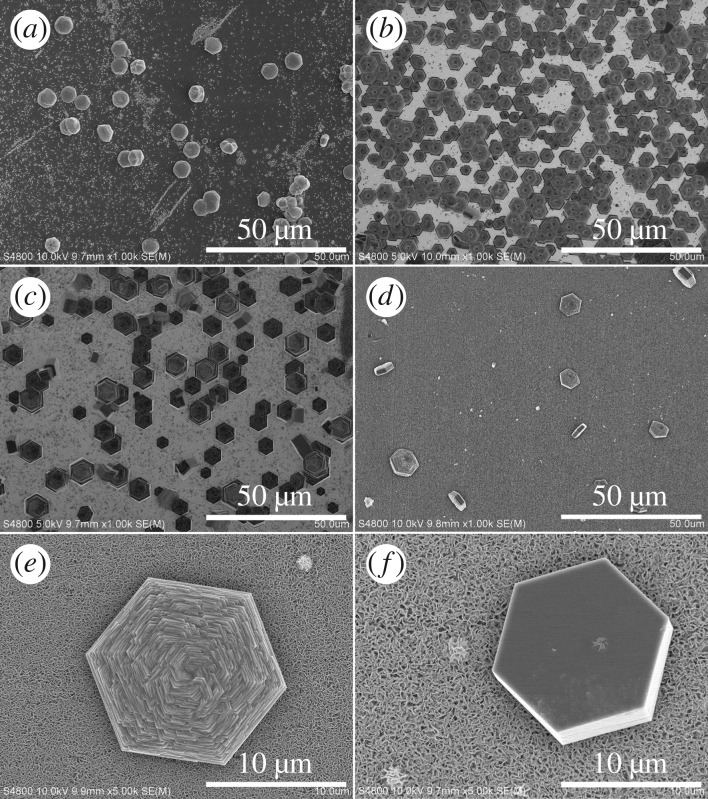
SEM images of the ZnO microdiscs obtained by the solution method at 95°C for 7 h with different NH_4_F concentrations: (*a*) 0 M, (*b*) 0.01 M, (*c*) 0.03 M and (*d*) 0.05 M, (*e,f*) The images of top surface and bottom surface of a ZnO microdisc obtained with 0.03 M NH_4_F.

The SEM images of ZnO microdiscs obtained at 95°C for 7 h with different NH_4_F concentrations in the nutrient solution are shown in figure 1*a–d*. As sodium citrate is provided in the precursor solution acting as the capping agent to selectively inhibit ZnO growth along the c-axis, near-round shaped ZnO microdiscs with approximately 7.5 µm in diameter are obtained without the addition of NH_4_F, as shown in figure 1*a*. The ZnO microdiscs are randomly distributed on the substrate without preferential orientation. Some of them are vertically grown on the silicon substrate. This is consistent with the reported results in the literature [27–30]. As an acidic mineralizer, NH_4_F has been reported to increase the crystallinity and crystal size of various materials grown by the solution method [22,31–33]. In this work, it is obvious to figure out that NH_4_F greatly affects the growth of ZnO microdiscs. Highly regular shape ZnO microdiscs with clear hexagonal edges were obtained with the addition of NH_4_F, as shown in figure 1*b–d*. Most ZnO microdiscs were uniformly distributed on the silicon surface along with ZnO c-axis. When the amount of NH_4_F is small (0.01 M), the shape of ZnO microdiscs changes from near-round to hexagonal, along with a notable change in their diameters. By raising the concentration of NH_4_F to 0.03 M, the diameter of the ZnO microdiscs is increased and ZnO microdiscs with diameter as large as 16 µm can be obtained. As shown in figure 1*e–f*, the top surface of ZnO microdiscs presents a multi-layered structure, while the bottom surface is smooth. However, further increasing the concentration of NH_4_F to 0.05 M, the distribution of the microdiscs on substrate became very sparse and some of them are also vertically grown on the silicon substrate. The thickness of the ZnO microdiscs adding 0, 0.01, 0.03 and 0.05 M is 1.5, 2, 7 and 4 µm, respectively, as shown in electronic supplementary material, figure S1. The changes in the morphology, density and diameter of ZnO microdiscs indicate that NH_4_F has really played an important role in the growth of ZnO nanostructures. As an acidic mineralizer, NH_4_F not only promotes the growth of ZnO nanostructure along the polar, semipolar and nonpolar faces but also remarkably increases their growth rates and enhances the crystallization quality [19,22]. The solubility of the precursors can be promoted significantly and the chemical potential of the solution could be increased through the addition of F^−^ which would be more favourable for nanostructure growth. F^−^ also can decrease the solution viscosity, and therefore make the mobility of ions improved [22]. The growth reactions are similar to those reported in the literature [34,35], as shown in equations (3.1) and (3.2).

3.1 $${\left(\text{C}{\text{H}}_{2}\right)}_{6}{\text{N}}_{4}+10{\text{H}}_{2}\text{O}\to \text{6HCHO(gas) + 4N}{\text{H}}_{4}^{+}\;+\;4OH^{-}$$

3.2 $$\text{2O}{\text{H}}^{-}+Z{\text{n}}^{2+}\to \text{Zn}{\left(\text{OH}\right)}_{2}\;\overset{\Delta }{⟶}\text{ZnO(s) + }{\text{H}}_{2}\text{O}.$$

**Figure 2.**
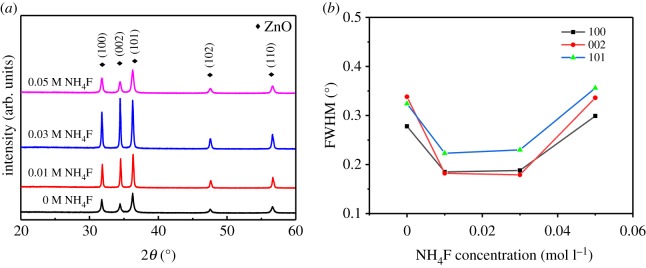
(*a*) XRD patterns of ZnO microdiscs synthesized by the solution method for 7 h with different NH_4_F concentrations (*b*) FWHM of XRD peaks of ZnO microdiscs synthesized with different NH_4_F concentrations.

**Figure 3.**
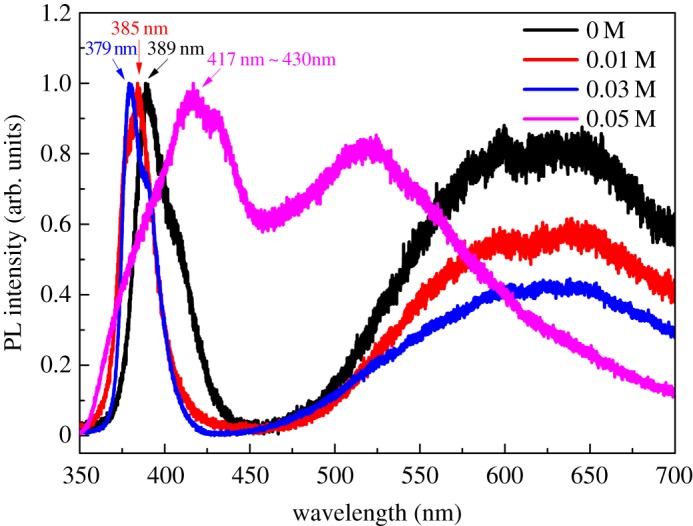
Room-temperature PL spectra of the as-prepared ZnO microdiscs with 0 M (black line), 0.01 M (red line), 0.03 M (blue line) and 0.05 M NH_4_F (purple line).

In these equations, equation (3.1) dominates the whole reaction. As is known, the surfactant sodium citrate can remarkably inhibit the growth of ZnO along c-axis direction, thus resulting in the appearance of round ZnO microdiscs, as shown in figure 1*a*. While adding NH_4_F, the growth rates of ZnO crystals are greatly promoted except for the c-axis direction, so it leads to larger hexagonal ZnO microdiscs with clear edges and multi-layered structures as shown in figure 1*b,c*. However, excessive NH_4_F (0.05 M) would apparently decrease the crystal size and suppress the yield of ZnO microdiscs, which is probably due to the fact that a large amount of added NH_4_^+^ ions might hinder the HMT hydrolysis to produce OH^−^ (equation (3.1)) and promotes the formation of ZnF(OH) due to the excess F^−^, all of which would impede the crystallization of ZnO nuclei [36]. X-ray photoelectron spectroscopy (XPS) measurement was conducted to determine the F-doping concentration. The results are shown in electronic supplementary material, figure S2. The calculated F concentration for 0.01, 0.03 and 0.05 M samples is 4.3%, 6.3% and 17%, respectively. As we know that XPS explores the surface information, energy-dispersive spectroscopy (EDS) was also performed to check the F ion concentration. The data in the supplement information show that for 0.03 and 0.05 M samples, the F ion concentrations are 4% and 9%, respectively. Owing to the low sensitivity of F in EDS, we have not got the F signal in 0.01 M sample. These results are close to the presupposed concentrations, which are 1%, 3% and 5% for 0.01, 0.03 and 0.05 M NH_4_F doping, respectively. This result also shows that the F^−^ ions prefer to be absorbed on the surface of ZnO. Because the F concentration obtained from XPS, which shows almost the surface status, is larger than that from EDS, which interprets the bulk information. Owing to the large electronegativity, the absorbed F atoms can eliminate the oxygen vacancy, thus improving the crystal quality. The effects of NH_4_F on the crystallinity of the ZnO microdiscs are further investigated through XRD and PL measurements. The results are shown in figures 2 and 3, respectively.

Figure 2*a* shows the XRD patterns of the samples grown with different NH_4_F concentrations. As depicted in figure 2*a*, the XRD patterns are indexed as the typical hexagonal wurtzite-type ZnO and no diffraction peaks from impurities can be found. Although all samples have similar XRD patterns, the full width at half maximum (FWHM) of peaks belonging to (100), (002) and (101) planes vary with concentration of the additive NH_4_F. It can be seen clearly from figure 2*b* that the FWHM of XRD peaks first decreased with NH_4_F concentration. At 0.03 M NH_4_F, the FWHM reaches the smallest value among the samples, indicating the highest crystallinity. When the concentration of NH_4_F additive reaches 0.05 M, the quality of crystallization becomes retrogressed, which is in accord with the SEM results observed in figure 1. This result proves that proper concentration of NH_4_F additive can promote crystallization and improve crystalline quality, as we mentioned before.

Photoluminescence measurement is used to investigate the defect existing in as-grown ZnO. Figure 3 shows the normalized PL spectra of ZnO microdiscs with different amounts of NH_4_F. Without adding NH_4_F, the main UV emission centres at 389 nm (near-band-edge (NBE) emission of ZnO) and the visible emission centres at 625 nm (related to oxygen interstitial (O_i_) and oxygen vacancy (V_O_) defects [37–39]). After the addition of NH_4_F, the UV emission presents a blue shift from 389 nm to 379 nm and the visible emission diminishes gradually with increased NH_4_F from 0 to 0.03 M. This means that the crystallinity of ZnO microdiscs gets better with the increase of NH_4_F concentration from 0 to 0.03 M. The blue shift of the UV peak in the PL spectra of ZnO microdiscs is probably attributed to the Burstein–Moss effect due to the *n*-type doping [38,40] by F^−1^ and the improvement of crystallinity and the reduction of defects. The defect energy levels have been filled up that make the NBE emission blue shifts. The weakening of visible emission delivers other evidence for enhanced crystal quality. However, further increasing the concentration of NH_4_F to 0.05 M would result in both changes of the peak position in the near UV and visible emission due to the superfluous F^−^ that participated in chemical etching. The blue to green light emission was enhanced, while UV and yellow to red emissions were suppressed. The similar phenomenon was reported before in the literature where the researchers found absorption peaks at 455 and 586 nm in their absorption spectra of F-doped ZnO samples [39]. Although F^−^ can passivate V_O_ defect due to large electronegativity which can lead to the suppression of V_O_-related emissions, excess F^−^ absorbed on the surface may induce other defect energy levels. This destroys the surface of ZnO microdiscs and increases defects. Although there is no impurity phase in the XRD spectrum, the excess F^−^ might be absorbed to the surface of the microdiscs forming defect states, resulting in the poor optical properties.

**Figure 4.**
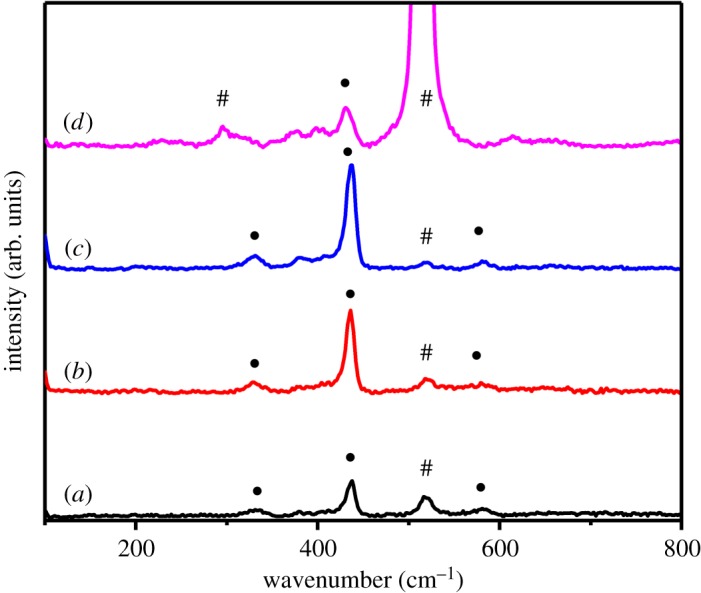
Raman spectra obtained from ZnO microdiscs grown with 0 M (black line), 0.01 M (red line), 0.03 M (blue line) and 0.05 M (purple line) NH_4_F. • and # denote the Raman vibration peaks corresponding to the ZnO and Si, respectively.

As shown in figure 4, all Raman spectra obtained from ZnO microdiscs with different amounts of NH_4_F exhibit the main peaks at 436.71 cm^−1^ attributed to the $E_{2}^{\mathrm{high}}$ vibration mode of ZnO, though the intensities have some variations [41]. The peaks at 437, 332, 380 and 584 cm^−1^ of ZnO microdiscs correspond to $E_{2}^{\mathrm{high}}$, $E_{2}^{\mathrm{high}}-E_{2}^{\mathrm{low}}$, A_1_ (TO) and E_1_ (LO) mode, respectively [42]. The peak at 520 cm^−1^ originates from Si substrate. The intensities of $E_{2}^{\mathrm{high}}$ band of Raman spectra are strengthened along with the increase of NH_4_F concentration from 0 to 0.03 M, suggesting that the NH_4_F additive plays an important role in promoting crystal growth and crystallization improvement. However, the decreased intensity of $E_{2}^{\mathrm{high}}$ band with 0.05 M NH_4_F may be caused by the deteriorated crystal quality, which is proved by XRD and PL measurements results discussed below. Hence, we have found that with 0.03 M NH_4_F additive, the relatively large and regular hexagonal ZnO microdisc can be achieved, with better crystal quality and lower defect concentration. Thus, a ZnO microdisc/*p*-Si photodetector has been fabricated through a simple transfer method for demonstration.

**Figure 5.**
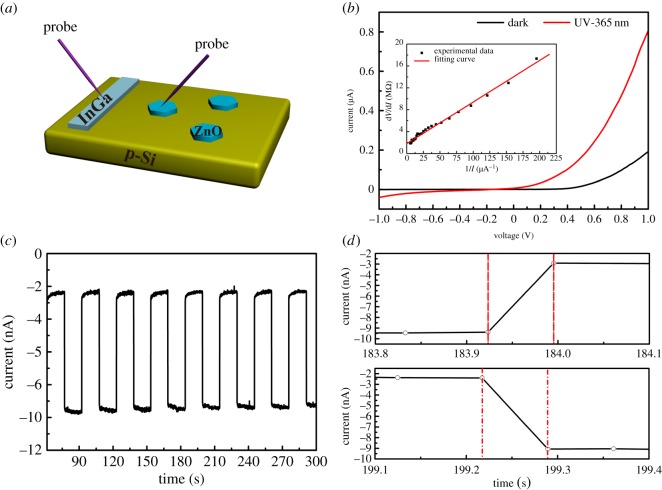
(*a*) Schematic illustration of the fabricated ZnO microdisc/*p*-Si UV detector. (*b*) *I–V* curves of the device in the dark and under the illumination of UV light (365 nm). The inset is the fitting of ideality factor *n*. (*c*) The response curve of the device operated at −0.6 V bias. (*d*) The response and recovery time of the device from magnified part of the response curve of figure 5(*c*).

Figure 5*a* shows the schematic illustration of the as-fabricated *n-*ZnO microdisc/*p*-Si UV detector. The probe was directly contacted onto the microdisc as the cathode and the InGa alloy was scraped onto the *p*-Si substrate as the anode. Figure 5*b* depicts the *I–V* characteristics of the detector under dark and UV irradiation (365 nm, approx. 4.1 mW cm^−2^). Without light illumination, the device reveals good rectification ratio of about 245 at ± 1 V, which is better than those of *n*-ZnO nanorod/*p*-Si devices reported in the literature [43]. When the UV light (365 nm) illuminated the device, the current increases and the device show a sensitivity of 400%, which is calculated by the following equation:

3.3 $$S(\text{\%})=\frac{I_{\mathrm{light}}+I_{\mathrm{dark}}}{I_{\mathrm{dark}}}\times 100,$$

where, *I*_light_ and *I*_dark_ are the currents measured with and without the UV light (365 nm) illumination, respectively. The ideality factor *n* of the heterojunction device is calculated to be 2.93, according to the method reported in the literature, as shown in the inset of figure 5*b* [44]. The ideality factor of this device is larger than that of an ideal *p*-*n* junction, which may be due to the imperfect interfaces between ZnO microdiscs and *p*-Si. Figure 5*c* depicts response curve of the device operating at −0.6 V bias with UV light on/off, suggesting good reproducibility. Under UV irradiation, the photocurrent increases to a stable value of approximately 10 nA. When the UV light turns off, the photocurrent drops down to the initial value of approximately 2 nA rapidly, the photoresponsivity of 0, 0.01 and 0.05 M samples is 1.19, 0.66 and 0.85 A W^−1^, respectively, at −1 V bias, as shown in electronic supplementary material, figure S3. Besides the stability, another important parameter to evaluate the performance of a photodetector is response speed. Figure 5*d* shows the response and recovery characteristic of the device. It is easy to figure out that the fast response and recovery time are less than 0.1 s, accompanying slow response and recovery component. Through exponent fitting, we can deduce that the slow response time is 12.92 s and the slow recovery time is 3.58 s. The rather short response and recovery time benefit from the improved crystal quality. The slow response and recovery parts originate from the oxygen absorbing and desorbing process [45,46]. The UV detector can reach a relatively large photoresponsivity of 1.19 A W^−1^ at −0.6 V bias, which is better than the reported ZnO nanorod photodetector [47]. The spectral photoresponsivity in electronic supplementary material, figure S4 shows a relatively high response in the UV region. This relatively high response originates from the ZnO microdiscs [48].

## 4. Conclusion

In summary, large hexagonal ZnO microdiscs were synthesized through a simple and low-energy-cost one-pot solution method with the aid of ammonium fluoride (NH_4_F). The growth rate and crystal quality of ZnO hexagonal microdiscs can be improved with proper NH_4_F concentration. ZnO microdiscs grown as large as 16 µm in diameter with 0.03 M NH_4_F can be achieved. Enhanced UV emission was obtained and the visible emission was suppressed with proper concentration of NH_4_F. The blue to green light emission of the ZnO disc was enhanced, while UV and yellow to red emissions were suppressed with further addition of NH_4_F. Based on the results, an ultraviolet *n-*ZnO microdisc/*p*-Si photodetector with a good rectifying characteristic (approx. 245 at ± 1 V) has been fabricated. The response and recovery time of the device is less than 0.1 s, under ultraviolet illumination (4.1 mW cm^−2^). The photoresponsivity can reach a relatively large value of 1.19 A W^−1^, with power consumption less than 10 nW. This kind of n-ZnO microdisc/*p*-Si device may be useful in highly efficient low power consumption photodetector applications.

## Supplementary Material

Supplementary material

## Data accessibility

All data generated or analysed during this study are included in this article.

## References

[1] Zhang Z, Wang SJ, Yu T, Wu T 2007 Controlling the growth mechanism of ZnO nanowires by selecting catalysts. J. Phys. Chem. C 111, 17 500–17 505. (10.1021/jp075296a)

[2] Sun Y, Riley DJ, Ashfold MNR 2006 Mechanism of ZnO nanotube growth by hydrothermal methods on ZnO film-coated Si substrates. J. Phys. Chem. B 110, 15 186–15 192. (10.1021/jp062299z) 16884233

[3] Zhang XY, Dai JY, Ong HC, Wang N, Chan HLW, Choy CL 2004 Hydrothermal synthesis of oriented ZnO nanobelts and their temperature dependent photoluminescence. Chem. Phys. Lett. 393, 17–21. (10.1016/j.cplett.2004.06.012)

[4] Wang Y, Li X, Wang N, Quan X, Chen Y 2008 Controllable synthesis of ZnO nanoflowers and their morphology-dependent photocatalytic activities. Sep. Purif. Technol. 62, 727–732. (10.1016/j.seppur.2008.03.035)

[5] Özgür Ü, Alivov YI, Liu C, Teke A, Reshchikov MA, Doğan S, Avrutin V, Cho S-J, Morkoç H 2005 A comprehensive review of ZnO materials and devices. J. Appl. Phys. 98, 11 (10.1063/1.1992666)

[6] Pearton SJ, Norton DP, Ip K, Heo YW, Steiner T 2004 Recent advances in processing of ZnO. J. Vac. Sci. Technol. B 22, 932–948. (10.1116/1.1714985)

[7] Guo H, Zhang W, Sun Y, Zhou T, Qiu Y, Xu K, Zhang B, Yang H 2015 Double disks shaped ZnO microstructures synthesized by one-step CTAB assisted hydrothermal methods. Ceram. Int. 41, 10 461–10 466. (10.1016/j.ceramint.2015.04.122)

[8] Rai P, Raj S, Lee I-H, Kwak W-K, Yu Y-T 2013 Conversion of ZnO microrods into microdisks like structures and its effect on photoluminescence properties. Ceram. Int. 39, 8287–8291. (10.1016/j.ceramint.2013.03.098)

[9] Self K, Zhou H, Greer HF, Tian ZR, Zhou W 2013 Reversed crystal growth of ZnO microdisks. Chem. Commun. 49, 5411–5413. (10.1039/C3CC41208C) 23660621

[10] Chen R, Ling B, Sun XW, Sun HD 2011 Room temperature excitonic whispering gallery mode lasing from high-quality hexagonal ZnO microdisks. Adv. Mater. 23, 2199–2204. (10.1002/adma.201100423) 21462376

[11] Xu CX, Sun XW, Dong ZL, Cui YP, Wang BP 2007 Nanostructured single-crystalline twin disks of zinc oxide. Cryst. Growth Des. 7, 541–544. (10.1021/cg060642j)

[12] Yousefi R, Zak AK, Mahmoudian MR 2011 Growth and characterization of Cl-doped ZnO hexagonal nanodisks. J. Solid State Chem. 184, 2678–2682. (10.1016/j.jssc.2011.08.001)

[13] Qin N, Xiang Q, Zhao H, Zhang J, Xu J 2014 Evolution of ZnO microstructures from hexagonal disk to prismoid, prism and pyramid and their crystal facet-dependent gas sensing properties. Cryst. Eng. Commun. 16, 7062–7073. (10.1039/C4CE00637B)

[14] Dai J, Xu CX, Xu XY, Li JT, Guo JY, Lin Y 2013 Controllable fabrication and optical properties of Sn-doped ZnO hexagonal microdisk for whispering gallery mode microlaser. APL Mater. 1, 032105 (10.1063/1.4820432)

[15] Dong H, Yang Y, Yang G 2014 Directional emission from ZnO hexagonal disks. ACS Appl. Mater. Interfaces 6, 3093–3098. (10.1021/am4058869) 24552159

[16] Li Z, Qin W, Wu XH 2015 Controllable hydrothermal synthesis of Al-doped ZnO with different microstructures, growth mechanisms, and gas sensing properties. RSC Adv. 5, 56 325–56 332. (10.1039/C5RA06233K)

[17] Li J, Mao Y, Cao W, Sun L, Peng X 2015 Benzenedicarboxylic acid-assisted synthesis of ZnO micro-hexagons from zinc hydroxide nanostrands and their photoluminescence properties. Appl. Phys. A 118, 683–690. (10.1007/s00339-014-8780-x)

[18] Choi K, Kang T, Oh S-G 2012 Preparation of disk shaped ZnO particles using surfactant and their PL properties. Mater. Lett. 75, 240–243. (10.1016/j.matlet.2012.02.031)

[19] Bao Q et al. 2013 Ammonothermal crystal growth of GaN using an NH**_4_**F mineralizer. Cryst. Growth Des. 13, 4158–4161. (10.1021/cg4007907)

[20] Bao Q et al. 2014 Ammonothermal growth of GaN on a self-nucleated GaN seed crystal. J. Cryst. Growth 404, 168–171. (10.1016/j.jcrysgro.2014.06.052)

[21] Schimmel S et al. 2015 Determination of GaN solubility in supercritical ammonia with NH**_4_**F and NH**_4_**Cl mineralizer by *in situ* x-ray imaging of crystal dissolution. J. Cryst. Growth 418, 64–69. (10.1016/j.jcrysgro.2015.02.020)

[22] Wang Z, Lu Y, Yuan S, Shi L, Zhao Y, Zhang M, Deng W 2013 Hydrothermal synthesis and humidity sensing properties of size-controlled zirconium oxide (ZrO**_2_**) nanorods. J. Colloid Interface Sci. 396, 9–15. (10.1016/j.jcis.2012.12.068) 23411357

[23] Zhang X, Zhu L, Xu H, Chen L, Guo Y, Ye Z 2014 Highly transparent conductive F-doped ZnO films in wide range of visible and near infrared wavelength deposited on polycarbonate substrates. J. Alloys Compd. 614, 71–74. (10.1016/j.jallcom.2014.06.098)

[24] Cao L, Zhu L, Jiang J, Zhao R, Ye Z, Zhao B 2011 Highly transparent and conducting fluorine-doped ZnO thin films prepared by pulsed laser deposition. Sol. Energy Mater. Sol. Cells 95, 894–898. (10.1016/j.solmat.2010.11.012)

[25] Guo Y, Zhu L, Li Y, Niu W, Zhang X, Ye Z 2015 Interaction of H and F atoms—origin of the high conductive stability of hydrogen-incorporated F-doped ZnO thin films. Thin Solid Films 589, 85–89. (10.1016/j.tsf.2015.05.003)

[26] Guillén-Santiago A, Olvera ML, Maldonado A, Asomoza R, Acosta DR 2004 Electrical, structural and morphological properties of chemically sprayed F-doped ZnO films: effect of the ageing-time of the starting solution, solvent and substrate temperature. Physica Status Solidi A 201, 952–959. (10.1002/pssa.200306727)

[27] Gang MG, Shin SW, Gurav K V, Wang YB, Agawane G, Lee JY, Moon J-H, Kim JH 2013 Studies on the controlling of the microstructural and morphological properties of Al doped ZnO thin films prepared by hydrothermal method. Jpn. J. Appl. Phys. 52, A06 (10.7567/JJAP.52.10MA06)

[28] Sisman I, Can M, Ergezen B, Bicer M 2015 One-step anion-assisted electrodeposition of ZnO nanofibrous networks as photoanodes for dye-sensitized solar cells. RSC Adv. 5, 73 692–73 698. (10.1039/C5RA13623G)

[29] Khoa NT, Kim SW, Van TD, Yoo D-H, Kim EJ, Hahn SH 2014 Hydrothermally controlled ZnO nanosheet self-assembled hollow spheres/hierarchical aggregates and their photocatalytic activities. Cryst. Eng. Commun. 16, 1344–1350. (10.1039/C3CE41763H)

[30] Kim JH, Andeen D, Lange FF 2006 Hydrothermal growth of periodic, single-crystal ZnO microrods and microtunnels. Adv. Mater. 18, 2453–2457. (10.1002/adma.200600257)

[31] Caullet P, Paillaud J-L, Simon-Masseron A, Soulard M, Patarin J 2005 The fluoride route: a strategy to crystalline porous materials. C.R. Chim. 8, 245–266. (10.1016/j.crci.2005.02.001)

[32] Chen G, Jiang L, Wang L, Zhang J 2010 Synthesis of mesoporous ZSM-5 by one-pot method in the presence of polyethylene glycol. Microporous Mesoporous Mater. 134, 189–194. (10.1016/j.micromeso.2010.05.025)

[33] Mori K, Maki K, Kawasaki S, Yuan S, Yamashita H 2008 Hydrothermal synthesis of TiO**_2_** photocatalysts in the presence of NH**_4_**F and their application for degradation of organic compounds. Chem. Eng. Sci. 63, 5066–5070. (10.1016/j.ces.2007.06.030)

[34] Ashfold MNR, Doherty RP, Ndifor-Angwafor NG, Riley DJ, Sun Y 2007 The kinetics of the hydrothermal growth of ZnO nanostructures. Thin Solid Films 515, 8679–8683. (10.1016/j.tsf.2007.03.122)

[35] Long T, Yin S, Takabatake K, Zhnag P, Sato T 2008 Synthesis and characterization of ZnO nanorods and nanodisks from zinc chloride aqueous solution. Nanoscale Res. Lett. 4, 247 (10.1007/s11671-008-9233-2) 20596478 PMC2893838

[36] Saito N, Haneda H, Seo W-S, Koumoto K 2001 Selective deposition of ZnF(OH) on self-assembled monolayers in Zn-NH**_4_**F aqueous solutions for micropatterning of zinc oxide. Langmuir 17, 1461–1469. (10.1021/la000607t)

[37] Knutsen KE, Galeckas A, Zubiaga A, Tuomisto F, Farlow GC, Svensson BG, Kuznetsov AY 2012 Zinc vacancy and oxygen interstitial in ZnO revealed by sequential annealing and electron irradiation. Phys. Rev. B 86, 121203 (10.1103/PhysRevB.86.121203)

[38] Yang YH, Chen XY, Feng Y, Yang GW 2007 Physical mechanism of blue-shift of UV luminescence of a single pencil-like ZnO nanowire. Nano Lett. 7, 3879–3883. (10.1021/nl071849h) 18001107

[39] Kumar PMR, Kartha CS, Vijayakumar KP, Singh F, Avasthi DK 2005 Effect of fluorine doping on structural, electrical and optical properties of ZnO thin films. Mater. Sci. Eng. B 117, 307–312. (10.1016/j.mseb.2004.12.040)

[40] Shan FK, Liu GX, Lee WJ, Shin BC 2006 Stokes shift, blue shift and red shift of ZnO-based thin films deposited by pulsed-laser deposition. J. Cryst. Growth 291, 328–333. (10.1016/j.jcrysgro.2006.03.036)

[41] Singamaneni S, Gupta M, Yang R, Tomczak MM, Naik RR, Wang ZL, Tsukruk VV 2009 Nondestructive in situ identification of crystal orientation of anisotropic ZnO nanostructures. ACS Nano 3, 2593–2600. (10.1021/nn900687g) 19655727

[42] Cuscó R, Alarcón-Lladó E, Ibáñez J, Artús L, Jiménez J, Wang B, Callahan MJ 2007 Temperature dependence of Raman scattering in ZnO. Phys. Rev. B 75, 165202 (10.1103/PhysRevB.75.165202)

[43] Periasamy C, Chakrabarti P 2011 Large-area and nanoscale n-ZnO/p-Si heterojunction photodetectors. J. Vac. Sci. Technol. B 29, 051206 (10.1116/1.3628638)

[44] Long H, Li S, Mo X, Wang H, Huang H, Chen Z, Liu Y, Fang G 2013 Electroluminescence from ZnO-nanorod-based double heterostructured light-emitting diodes. Appl. Phys. Lett. 103, 123504 (10.1063/1.4821346)

[45] Safa S, Khajeh M, Azimirad R 2018 The effects of measuring atmosphere on ultraviolet photodetection performance of ZnO nanostructures. J. Alloys Compd. 735, 1406–1413. (10.1016/j.jallcom.2017.11.286)

[46] Safa S, Mokhtari S, Khayatian A, Azimirad R 2018 Improving ultraviolet photodetection of ZnO nanorods by Cr doped ZnO encapsulation process. Opt. Commun. 413, 131–135. (10.1016/j.optcom.2017.12.038)

[47] Chang SP, Lu CY, Chang SJ, Chiou YZ, Hsueh TJ, Hsu CL 2011 Electrical and optical characteristics of UV photodetector with interlaced ZnO nanowires. IEEE J. Sel. Top. Quantum Electron. 17, 990–995. (10.1109/JSTQE.2010.2046884)

[48] Azimirad R, Khayatian A, Safa S, Almasi Kashi M 2014 Enhancing photoresponsivity of ultra violet photodetectors based on Fe doped ZnO/ZnO shell/core nanorods. J. Alloys Compd. 615, 227–233. (10.1016/j.jallcom.2014.06.157)

