# Supplementary material for: NH4F-assisted one-pot solution synthesis of hexagonal ZnO microdiscs for efficient ultraviolet photodetection
Source: R Soc Open Sci. 2018 Sep 12;5(9):180822. doi: 10.1098/rsos.180822 (PMC6170531; doi:10.1098/rsos.180822)
Supplement: Supplementary material [file rsos180822supp1.doc]

**Supplementary material**

**NH_4_F-assisted one-pot solution synthesis of hexagonal ZnO microdiscs for efficient ultraviolet photodetection**

**Borui Li**‡**, Kai Zhou**‡**, Zhao Chen, Zengcai Song, Dong Zhang* and Guojia Fang***

*Key Lab of Artificial Micro- and Nano-Structures of Ministry of Education of China, School of Physics and Technology, Wuhan University, Wuhan 430072, People’s Republic of China*

Correspondence and requests for materials should be addressed to G.J.F. (email: gjfang@whu.edu.cn), D.Z. (email: dongz@whu.edu.cn)

‡These authors contributed to this work equally.

**
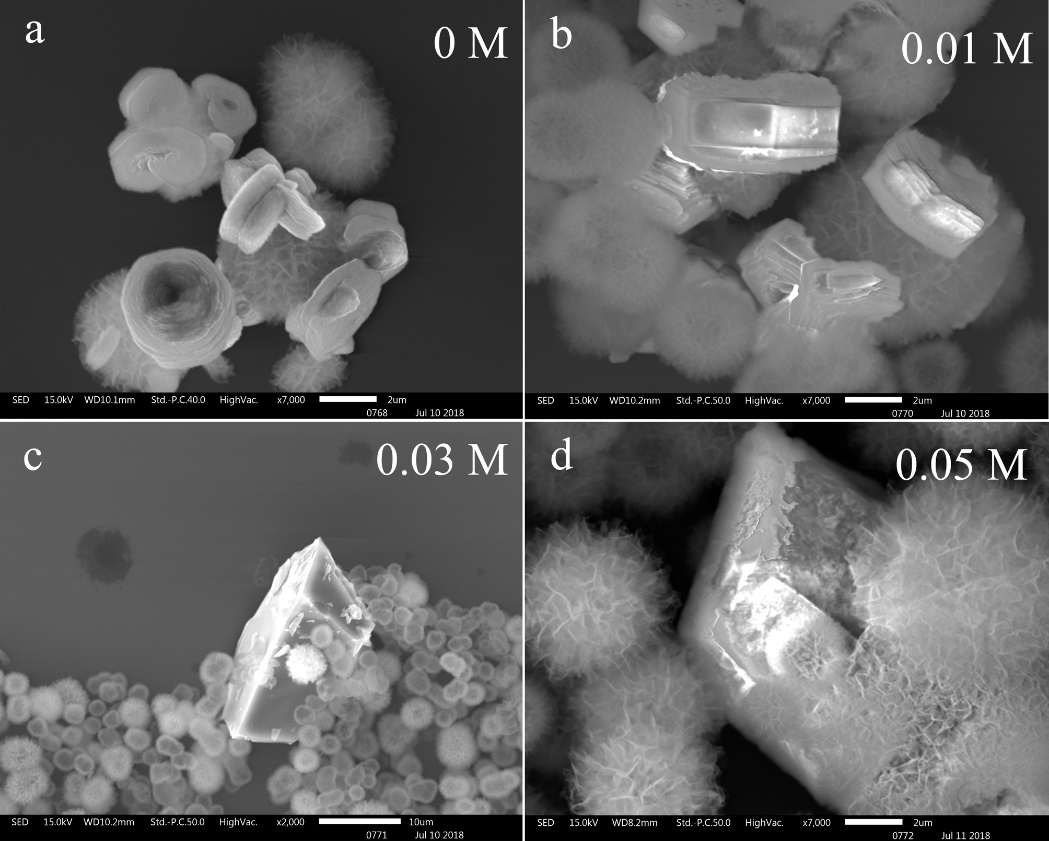
**

**Figure S1.** SEM for thickness measurement. a) 0 M NH_4_F. b) 0.01 M NH_4_F. c)0.03 M NH_4_F. d) 0.05 M NH_4_F

**
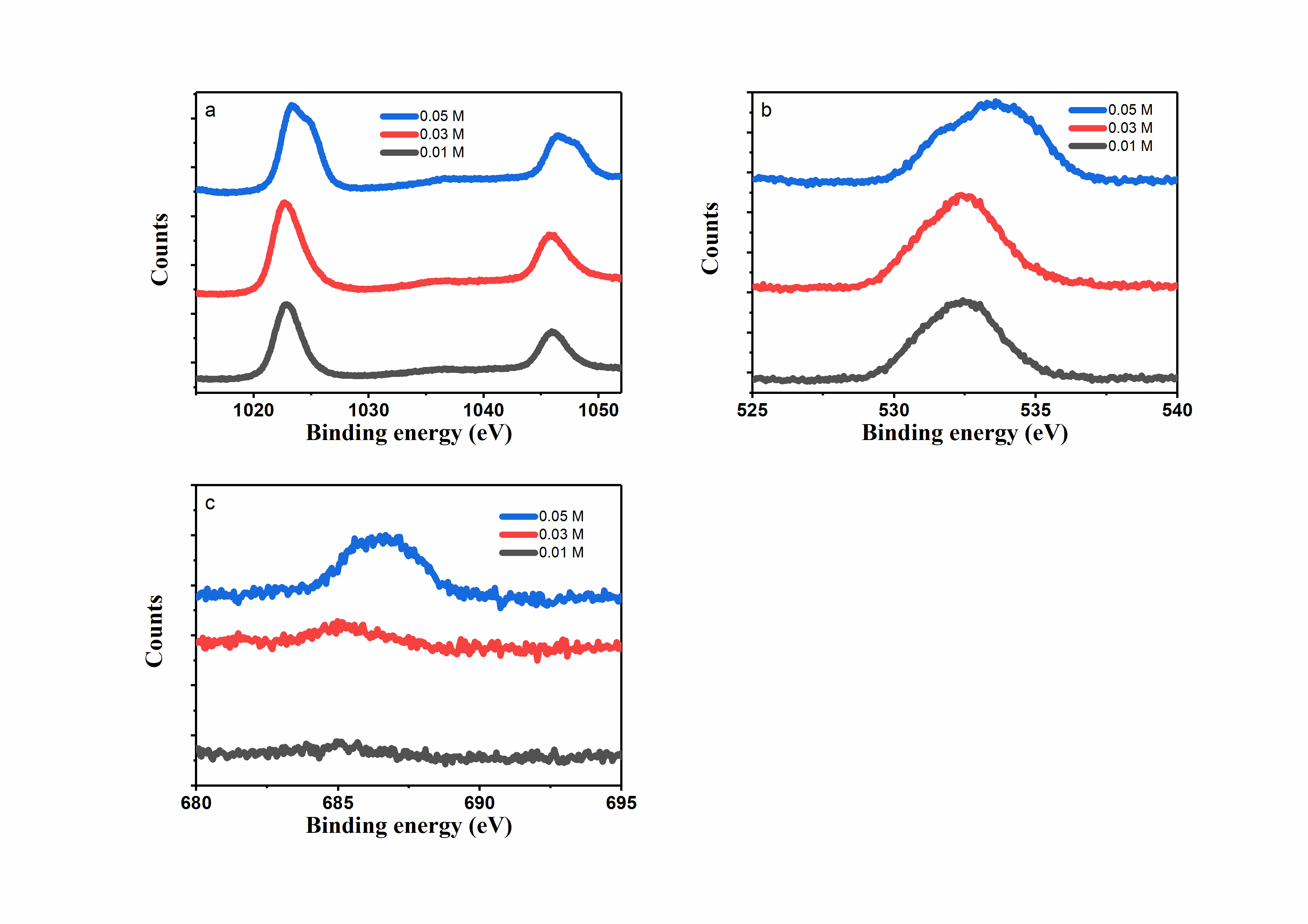
**

**Figure S2.** XPS of samples with 0.01 M, 0.03 M and 0.05 M NH_4_F. a) Zn 2p. b) O 1s. c) F 1s.

**
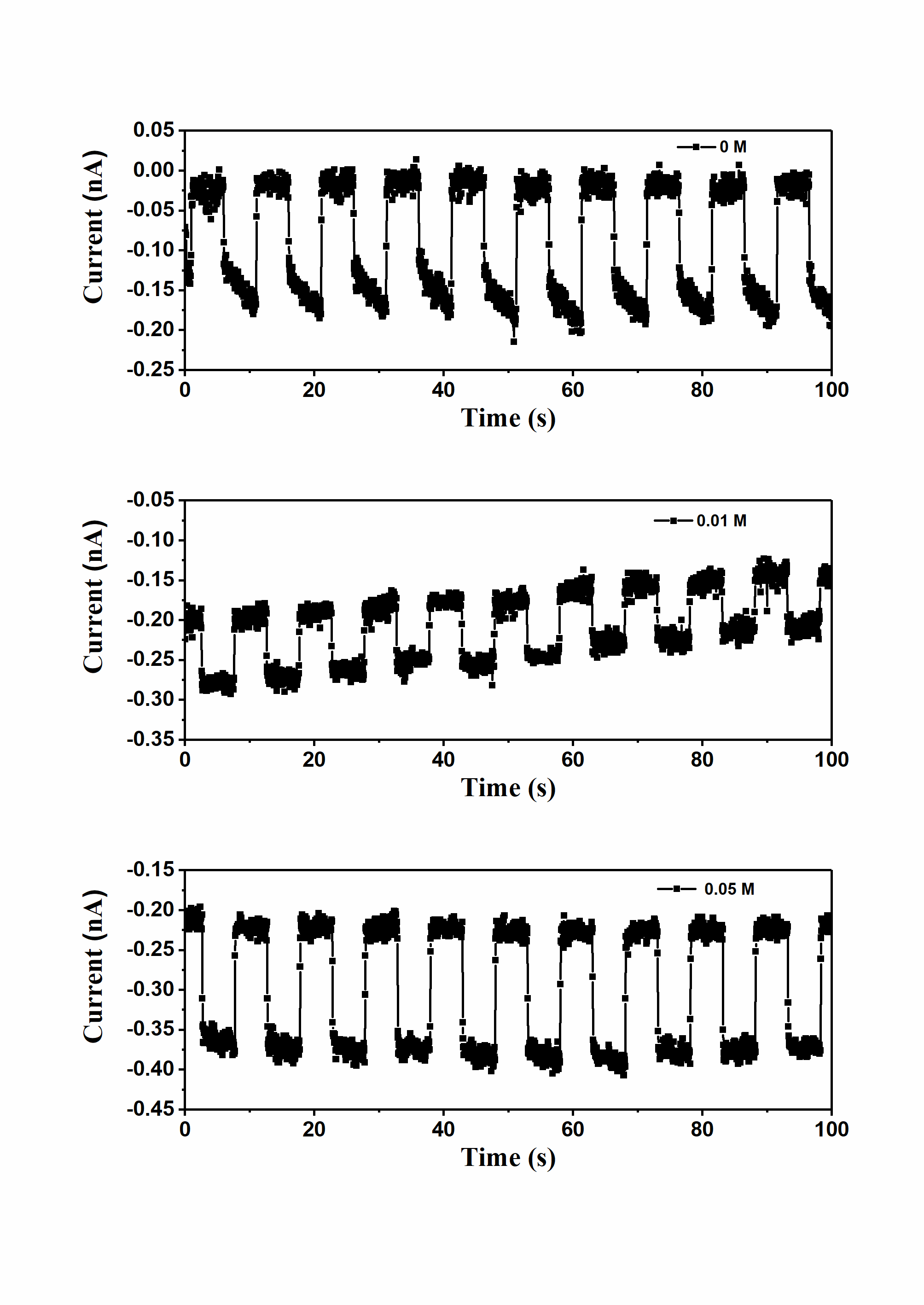
**

**Figure S3.** Photoresponse of the samples with different NH4F, measured at -1 V bias, with 365 nm illumination, 0.19 mW/cm^-2^.

**
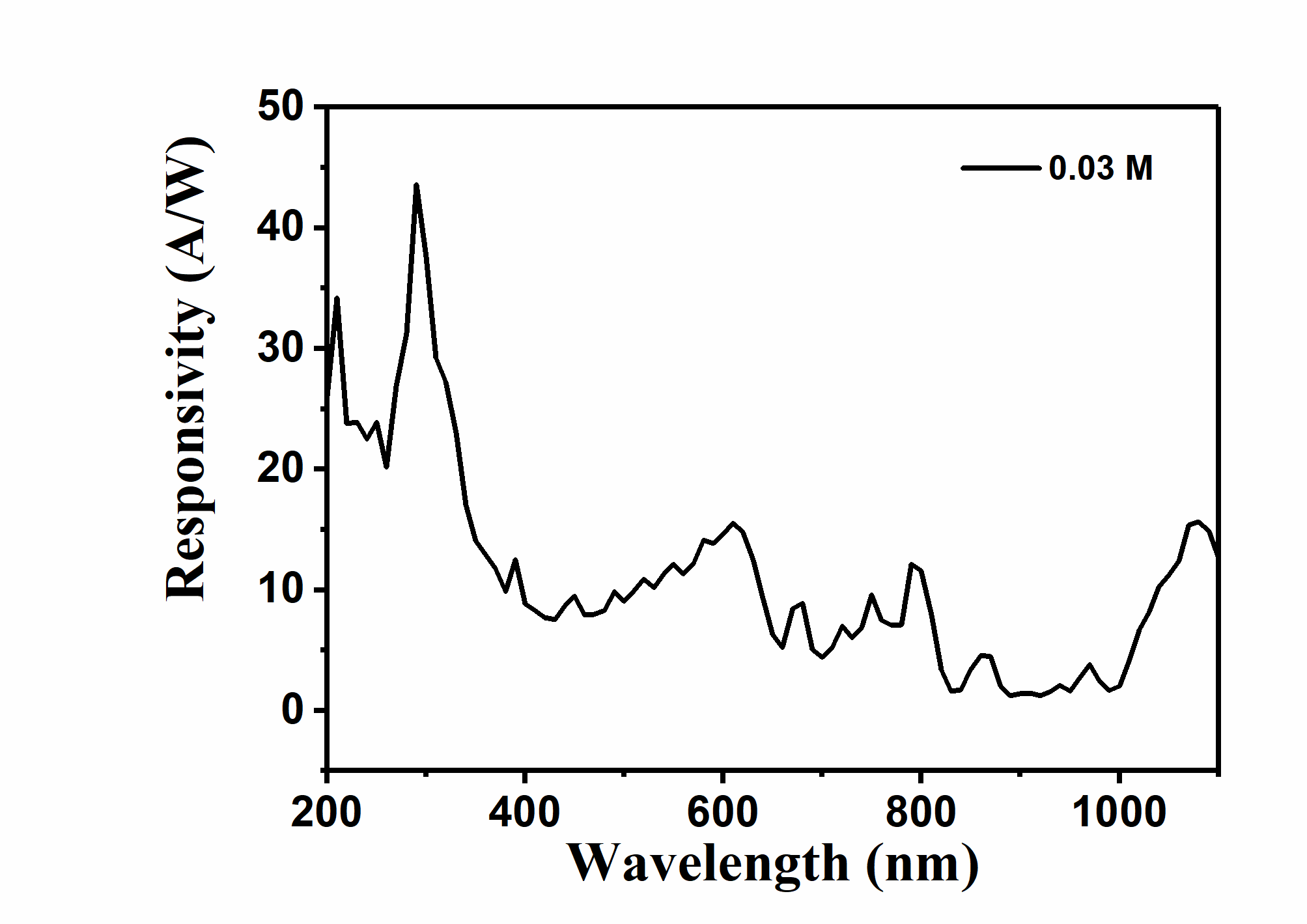
**

**Figure S4.** Spectral photoresponsivity of sample with 0.03 M NH_4_F.
